# Phylogeography reveals a potential cryptic invasion in the Southern Hemisphere of *Ceratophyllum demersum*, New Zealand’s worst invasive macrophyte

**DOI:** 10.1038/s41598-017-16712-8

**Published:** 2017-11-29

**Authors:** Benita Hyldgaard, Carla Lambertini, Hans Brix

**Affiliations:** 1 Department of Bioscience, Section of Aquatic Biology, Aarhus University, Aarhus, Denmark; 2 Department of Food Science, Section of Plants, Food and Climate, Aarhus University, Aarslev, Denmark

## Abstract

*Ceratophyllum demersum* (common hornwort) is presently considered the worst invasive submerged aquatic macrophyte in New Zealand. We explored the global phylogeographic pattern of the species, based on chloroplast and nuclear DNA, in order to identify the origin of the invasive populations in New Zealand and to clarify if there were multiple introductions. The phylogeographic study identified geographically differentiated gene pools in North America, tropical Asia, Australia, and South Africa, likely native to these regions, and a recent dispersal event of a Eurasian-related haplotype to North America, New Zealand, Australia, and South Africa. At least two different invasive genotypes of this Eurasian-related haplotype have been found in New Zealand. One genotype is closely related to genotypes in Australia and South Africa, while we could not trace the closest relatives of the other genotype within our *C. demersum* sample set. Contrasting spectra of genetic distances in New Zealand and in a region within the native range (Denmark), suggest that the invasive population was founded by vegetative reproduction, seen as low genetic distances among genotypes. We also discovered the introduction of the same Eurasian-related haplotype in Australia and South Africa and that a cryptic invasion may be occurring in these continents.

## Introduction

Global trade and transport has dramatically increased the number of alien plant species and populations introduced to novel areas either intentionally or accidentally by human activity^1,2^. Islands often have unique and fragile ecosystems with ecological niches that have not been filled because of the distance from colonizing populations^3,4^. Furthermore, the geographical isolation of islands, such as New Zealand, makes them particularly vulnerable to invasions because of the lack of natural competitors and predators that control populations in their native ecosystems, i.e. introduced populations are released from the enemies of their native ranges according to the enemy release hypothesis^5,6^. The spread of alien species into New Zealand and the development of some of those into invasive nuisance plants threatening the indigenous flora have occurred several times in the past century and are likely to still occur^7^. Freshwater ecosystems are more sensitive to invasions than terrestrial habitats^8^, and in New Zealand several aquatic macrophyte species such as *Ceratophyllum demersum* L., *Lagarosiphon major* (Ridley) Moss and *Egeria densa* Planch. are spreading across freshwater ecosystems at a great cost to the local ecosystems and economy^9^.

In some instances, introduced genotypes may have conspecific native populations in the invaded range and be morphologically similar to the native genotypes but sufficiently different in ecology or physiology to become invasive and outcompete the conspecific native populations as well as other species. This type of invasion is called cryptic, as the new invaders are difficult to distinguish from native populations due to their morphological resemblance^10^. These invasions are particularly difficult to recognize before changes in ecosystems are observed^10^.

*Ceratophyllum demersum* L. (common hornwort) is presently considered the worst invasive submerged macrophyte in New Zealand as it grows vigorously, forming surface-reaching canopies, and replaces native plant communities^3,11^. It is also an economic burden as it clogs hydrostations and prevents fishing and recreational activities in several lakes due to production of thick biomass mats^12^. *Ceratophyllum demersum* achieved a cosmopolitan distribution about 2.5 million years before present^13^. Given the fossil record, the species is considered native to North America, Eurasia, Africa and Australia^14–16^. In New Zealand, *C. demersum* was recorded for the first time in 1961 in the Hawke’s Bay region on the east coast of the North Island. Since then it has spread to many lakes and streams across the North Island^7^. In 2002, *C. demersum* was also found in two locations in the South Island, where it has now been eradicated.

In this study, samples of *C. demersum* were collected from all over the world to investigate the phylogeography of the species and trace the origin of the invasive populations in New Zealand. We also assessed the role of genetic differentiation in the invasion in New Zealand, i.e. if the invasion is driven by a population with high genetic diversity, and thus has potential for rapid evolution, or by one or few genotypes with high phenotypic plasticity^17^. We used both chloroplast DNA sequences (cpDNA sequences) to trace long-distance dispersal events, and nuclear amplified fragment length polymorphisms (AFLPs) to resolve intra-haplotypic relationships (i.e. between individuals sharing chloroplast DNA), within distribution ranges.

## Results

### cpDNA sequences

**Figure 1:**
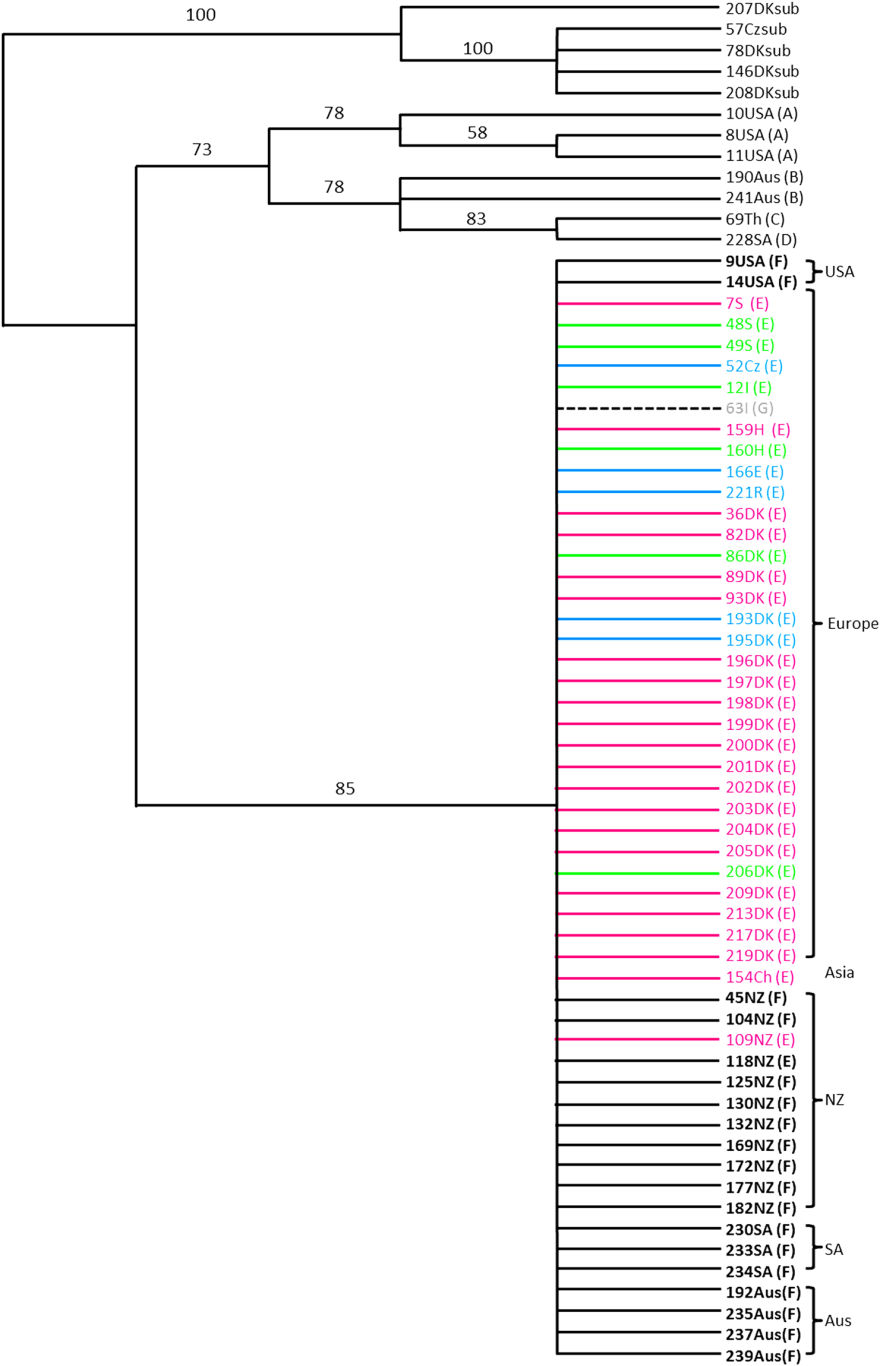
cpDNA regions (Cdemex 1 and rps16) consensus tree from jackknife analysis with 37% deletion (including cpDNA microsatellite loci). Black bold versus non-bold and dashed line indicate three different haplotypes differing in one base substitution within the global unresolved groups with 85% support. Haplotypes A-G are indicated in brackets after the sampleID. Different colours indicate variation of microsatellite loci within the European haplotype (incl. China, 154Ch and New Zealand, 109NZ).

In total, seven different haplotypes (Haplotype A-G, Fig. 1 and Supplementary Fig. S1) were identified by the cpDNA, within *C. demersum* (GenBank accession no. KJ093282- KJ093289 + KJ093291-KJ093300, Supplementary Table S1). The parsimony analysis (i.e. the simplest scientific explanation that fits the evidence) divided the haplotypes into two well-supported groups (Fig. 1 and Supplementary Fig. S1). One group included four haplotypes: the USA (US) (Haplotype A, GenBank accession no. KJ093282- KJ093284 and KJ093291- KJ093293, Cdemex 1 and rps16 respectively), Thailand (Th) (Haplotype C, GenBank accession no. KJ093287 and KJ093296), Australia (Aus) (Haplotype B, GenBank accession no. KJ093289 and KJ093300), and South Africa (SA) (Haplotype D, GenBank accession no. KJ093288 and KJ093299). The other group was represented by three closely related haplotypes (Haplotype E, F and G) differing in one substitution in the rps16 and one substitution in the Cdemex 1, and included most of our samples from the US, China (Ch), Aus, New Zealand (NZ), SA, and Europe (EU) independently from their geographic origin (Fig. 1). Of these three haplotypes, one haplotype (Haplotype G, GenBank accession no KJ093286 and KJ093295) was unique and found in one single sample collected in Italy (Trieste). The second haplotype (Haplotype E, GenBank accession no. KJ093285 Cdemex 1 and KJ093294 and KJ093297 rps16) was shared by all EU and Ch samples and showed variation at the microsatellite loci of the rps16 and Cdemex 1 regions. The third haplotype (Haplotype F, GenBank accession no. KJ093298, rps16) was shared by the samples from the US, NZ, Aus, and SA and did not show microsatellite variation in the two cpDNA regions studied. The phylogeographic structure remained unchanged when the microsatellite variation was omitted from the sequences (Supplementary Fig. S1). The microsatellite variation increased the geographic resolution within the first group and divided the US samples from those from Th, Aus, and SA samples (78% support for both groups), and suggested a closer relationship of the Th sample to the SA samples (83% support) rather than to the Aus samples (Fig. 1). *Ceratophyllum demersum* was separated from *C. submersum* in both trees with 100% support.

### AFLPs

**Figure 2:**
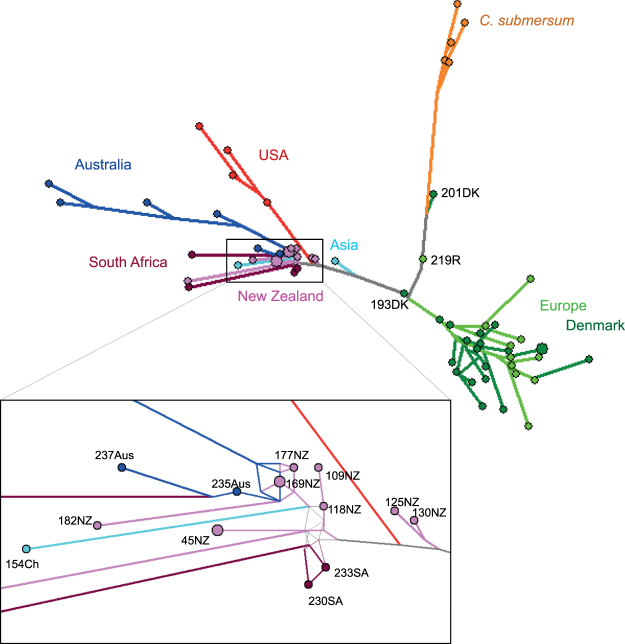
Median-joining network of AFLP data. The different colours indicate different geographic regions or different taxa. *Ceratophyllum submersum* (orange) and *C. demersum*: Europe (light green, Denmark: dark green), Asia (light blue), Australia (dark blue), New Zealand (purple), South Africa (dark red), and USA (red).

The AFLP median-joining network divided the samples into three major groups: (1) *C. submersum*, (2) EU, and (3) the rest of the world, being the US, tropical Asia (Th and Ch), Aus, SA, and NZ in our sample set (Fig. 2). In agreement with the cpDNA sequences, the US and some Aus samples appeared as two well-resolved branches within the third group. The samples from NZ split into different branches with unresolved connections mostly to the Aus and SA nodes. No phylogeographic structure was detected within the EU group, or among the Danish (DK) samples (dark green nodes in Fig. 2). Two *C. demersum* EU samples appeared to belong to the *C. submersum* branch.

**Figure 3:**
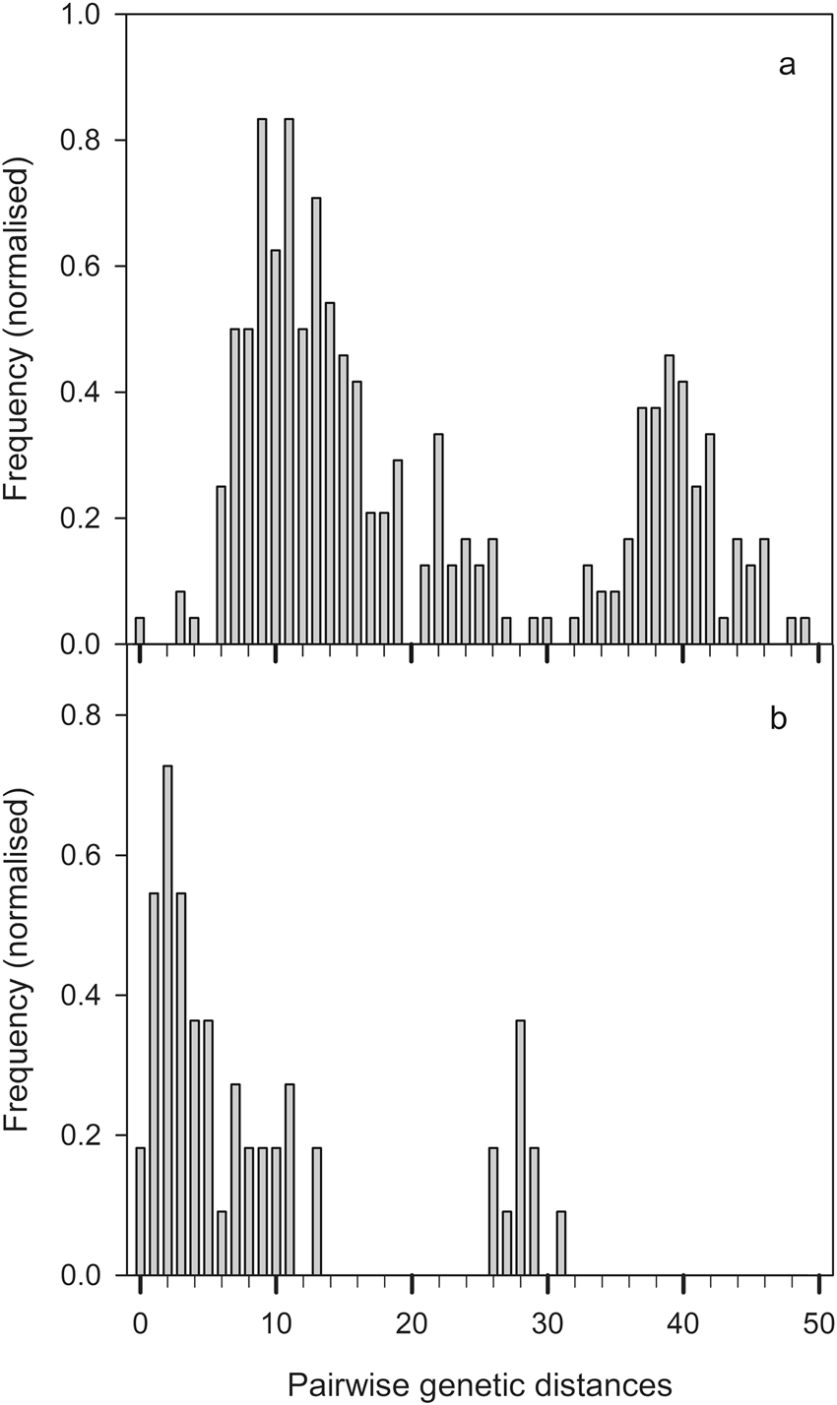
Spectra of pairwise genetic distances (**a**) between Danish *C. demersum* and *C. submersum*, (**b**) within New Zealand *C. demersum*. In (**b**) the pairwise genetic distances in the range 26–31 are between sample 132NZ and the rest of the NZ samples.

The spectra of genetic distances showed a similar discontinuous pattern with distinct groups of genetic distances in NZ and in the native range in DK (Fig. 3). The DK spectrum of pairwise genetic distances, which we used as a reference spectrum for interbreeding and non-interbreeding populations, split the genetic distances into two groups (Fig. 3a). The first group (range 0–30) showed a modal distribution and included pairwise genetic distances within *C. demersum* and within *C. submersum*. The second group (range 32–49) included pairwise genetic distances between *C. demersum* and *C. submersum*. Three samples of *C. demersum* showed similar genetic distances both to the other samples of *C. demersum* and to *C. submersum*. These may be regarded as an intermediate group of genetic distances (range 21–30) between the two species. One of these three samples was 201DK, which the network placed along the *C. submersum* branch, while the other two samples were included in the EU group (Fig. 2). The potential group of intermediate genetic distances between the two species prompted suspicion of morphologically undetectable interspecific hybrids might occasionally occur in *C. demersum* populations. Gene diversity (h) was 0.161 within the Danish *C. demersum* population and it decreased to 0.132 when the possible hybrids were omitted from the analysis (Table 1).

**Table 1:** AFLP gene diversity (**h**) (standard deviation in brackets) within haplotypes.

Phylo/geo subgroups within haplotypes	Species Haplotype	*C. sub*	*C. dem* A	*C. dem* B	*C. dem* C + D	*C. dem* E	*C. dem* F
	Geographic distribution	EU	US nat	Aus nat	Th-SA nat	EU-Asia	US-NZ-SA-Aus
	N						
*C. sub*	5	0.138 (0.019)					
*C. sub* DK	4	0.138 (0.020)					
US-NZ-SA-Aus	19						0.195 (0.014)
US nat	3		0.136 (0.021)				
US int	2						0.067 (0.017)
Aus nat	2			0.167 (0.023)			
Aus int	4						0.213 (0.021)
Aus int without Aus int _NZ_	2						0.110 (0.020)
Aus int _NZ_	2						0.024 (0.010)
NZ int incl. 132NZ	10						0.110 (0.014)
NZ int without 132NZ	9						0.069 (0.014)
NZ int without 132NZ and incl. 109NZ (E)	10						0.070 (0.014)
SA-Th nat	2				0.114 (0.021)		
SA int	3						0.121 (0.020)
SA int _NZ_	2						0.005 (0.005)
NZ + Aus int _NZ_ + SA int _NZ_	13						0.072 (0.013)
EU-AS	33					0.208 (0.017)	
EU incl. DK	31					0.194 (0.017)	
EU without DK	11					0.203 (0.018)	
DK	20					0.161 (0.017)	
DK without putative hybrids with *C. sub*	17					0.132 (0.018)	

(**N**) sample size. *C. submersum* (**C. sub**) and *C. demersum* (**C. dem**) haplotypes are identified by cpDNA sequences (**haplotypes A–F**). Phylogenetic and/or geographic groups are identified by cpDNA and AFLP data. The gene diversity values are indicated at the interception between phylogenetic/geographic groups and their cpDNA haplotype. The annotations “**nat**” (native), “**int**” (introduced), “**int**_**NZ**_” (introduced closely related to NZ genotypes) are tentative and added for clarity. This status has been deduced by combining the results of Figs 1 and 2 and Table 3. Geographic abbreviations in alphabetic order: **Aus** (Australia), **DK** (Denmark), **EU** (Europe), **NZ** (New Zealand), **SA** (South Africa), **Th** (Thailand), **US** (USA).

The spectrum of pairwise genetic distances within the NZ populations suggested two reproductively isolated *C. demersum* gene pools, given the bimodal and discontinuous distribution of genetic distances (Fig. 3b). The second peak included the genetic distances between the sample 132NZ (from Lake Tarawera) and the rest of the NZ samples, and revealed at least two discontinuously related genotypes and pointed to the possibility of two introductions. Compared with the spectrum of the DK populations, the NZ samples showed shorter pairwise genetic distances and a decreasing curve of genetic distances, whereas the genetic distances with sample 132NZ were of the same order of magnitude as between *C. demersum* and its suspected hybrids with *C. submersum* in DK. Gene diversity (h) was 0.110 in the NZ population and decreased to 0.069 when sample 132NZ was removed (Table 1). In an attempt to rule out the possibility of two morphologically similar *Ceratophyllum* species in NZ, we aligned our cpDNA sequences with those of *C. echinatum* and *C. submersum* in GenBank. Given the close AFLP relationship of the NZ samples to the US samples sharing the same haplotype, we assumed that *C. echinatum* might have contributed alleles to the gene pool of the US-Asian-Aus-NZ-SA branch of the network (Fig. 2). *Ceratophyllum echinatum* is morphologically similar to *C. demersum* and *C. submersum*. However, none of the NZ sequences matched the sequences of either *C. echinatum* or *C. submersum* in GenBank (matK and ITS).

Nei’s unbiased AFLP genetic identities showed that the most similar gene pools in our sample set to that of the NZ invasive population are in SA, Aus, and in Ch (for sample 172NZ), based on the pairwise genetic identities (Table 2). However, discontinuous pairwise genetic distances within these regions indicated two distantly related gene pools in SA and Aus (Table 3). The NZ samples had the shortest genetic distances to samples in the south of SA and in the east coast of Aus, and such samples in SA and Aus had shorter genetic distances to each other than to the conspecific genotypes in SA and Aus (Table 3). AFLP gene diversity (h) increased to 0.072 for the population of the NZ samples and its shortest genetically distant genotypes in SA and Aus (Table 1).

**Table 2:** Nei’s unbiased genetic identity based in AFLP data among all New Zealand samples and the different sampled geographic regions.

Location	n	Nei’s unbiased genetic identity											Pairwise genetic distances (within pop)	
		45NZ	104NZ	109NZ	118NZ	125NZ	130NZ	132NZ	169NZ	172NZ	177NZ	182NZ	Range	Mode
USA	5	0.777	0.787	0.789	0.796	0.781	0.791	0.563	0.801	0.769	0.83	0.797	11–23	15
Europe (incl. DK)	32	0.754	0.749	0.755	0.74	0.727	0.739	0.694	0.745	0.741	0.733	0.77	0–39	11
Europe (excl. DK)	12	0.737	0.726	0.741	0.718	0.715	0.723	0.679	0.73	0.725	0.721	0.755	5–39	12
Denmark (DK)	20	0.754	0.75	0.756	0.74	0.724	0.736	0.684	0.742	0.736	0.731	0.769	0–32	11
South Africa	4	**0.889**	**0.882**	**0.893**	**0.882**	**0.84**	**0.855**	0.635	**0.869**	0.852	**0.886**	**0.833**	1–28	21
Australia	6	0.754	0.766	0.77	0.766	0.758	0.758	**0.731**	0.778	0.774	0.772	0.755	5–39	29
China	1	0.829	0.857	0.829	0.857	0.819	0.838	0.619	0.829	**0.857**	0.838	0.781	—	—
Thailand	1	0.743	0.714	0.743	0.733	0.752	0.771	0.552	0.724	0.695	0.733	0.733	—	—
*C. submersum*	5	0.485	0.496	0.46	0.475	0.509	0.484	0.514	0.485	0.498	0.481	0.499	6–24	15
New Zealand	11												11355	2

The highest identity of each New Zealand sample is highlighted in bold.

**Table 3:** Pairwise genetic distances between the New Zealand samples and all single samples from South Africa, Australia and Asia.

		Pairwise genetic distances																					
		New Zealand											South Africa				Australia						Thailand
Location	Sample	45NZ	104NZ	109NZ	118NZ	125NZ	130NZ	132NZ	169NZ	172NZ	177NZ	182NZ	228SA	**230SA**	**233SA**	234SA	190Aus	192Aus	**235Aus**	237Aus	239Aus	241Aus	69Th
South Africa	228SA	21	20	20	21	19	21	34	20	19	19	22	—										
	**230SA**	**3**	**3**	**5**	**4**	**8**	**6**	23	**3**	**3**	**3**	**9**	21	—									
	**233SA**	**2**	**2**	**3**	**2**	**5**	**4**	28	**2**	**2**	**3**	**10**	21	**1**	—								
	234SA	26	26	25	26	33	31	35	28	26	29	29	28	26	24	—							
Australia	190Aus	42	40	39	40	38	37	33	39	38	36	43	34	40	41	35	—						
	192Aus	29	32	31	31	32	30	27	32	32	32	34	33	31	30	29	22	—					
	**235Aus**	**4**	**4**	**4**	**5**	**10**	**7**	28	**3**	**3**	**2**	**9**	20	**3**	**4**	27	37	30	—				
	**237Aus**	**8**	**9**	**8**	**9**	**11**	**10**	32	**7**	**9**	**6**	**10**	21	**7**	**8**	29	39	29	**5**	—			
	239Aus	36	33	35	34	35	34	29	31	30	34	32	39	33	32	29	26	23	32	32	—		
	241Aus	22	19	22	18	23	21	32	19	16	18	21	23	21	21	31	35	29	19	19	25	—	
Thailand	69Th	17	16	17	15	18	15	35	17	15	18	16	24	16	15	28	37	27	17	21	33	22	—
china	154Ch	13	10	14	12	16	13	30	13	10	13	20	23	12	14	36	39	28	15	18	34	23	27

Numbers highlighted in bold indicate the lowest genetic distances.

The relationship of sample 172NZ from the most northern sampling locality in NZ with the Ch sample (Table 2) appeared weaker than the relationship of this sample with the SA and Aus samples (Table 3). We could not trace any close relatives of sample 132NZ collected in Lake Tarawera within our sample set.

## Discussion

### Phylogeography of Ceratophyllum demersum

The parsimony analyses of cpDNA sequences combined with AFLPs suggest the worldwide phylogeographic structure of *C. demersum* to consist of two layers (Fig. 1). The first layer is a relic of an ancient disjunct distribution marked by the well-resolved branches in the consensus trees of the US, Th, Aus and SA samples (Haplotype A–D), and the second layer is unstructured which is likely due to a recent dispersal event of two closely related haplotypes. One of these haplotypes is dominant in the modern Eurasian populations (Haplotype E) and the other has dispersed across North America, NZ, Aus and SA (Haplotype F). The median-joining network of nuclear AFLPs (Fig. 2) has a similar structure of well resolved and unresolved branches which further support the occurrence of ancient, likely native populations in the US and Aus (well resolved branches), and of undifferentiated, intercontinentally-wide spread populations of closely related genotypes, occurring in North America, NZ, Aus and SA (unresolved branches), likely resulting from recent dispersal. In the US and Aus, where the recently introduced genotypes are sympatric with the ancient native populations, the network suggests that gene exchange has occurred, as some genotypes of the introduced haplotypes are placed in the branches of the native haplotypes. Long-distance dispersal by birds is regarded as a viable explanation for widely disjunct aquatic plant distributions^13^. However, human-mediated dispersal, such as the aquarium trade, is also a well-documented source of exotic propagules in the recent invasion history of many aquatic species^18^ and may have facilitated recent and repeated dispersal events among continents, and contact among previously isolated populations as suggested by the present study.

### *C. demersum* in New Zealand

*Ceratophyllum demersum* is a popular ornamental plant for aquaria and was sold in NZ until it was banned in 1982^7^. In NZ, it shows typical invasive traits such as high growth rates and creation of monocultures^19,20^. In our study, at least two introduced genotypes of *C. demersum* were found in NZ. One of these was found in several lakes in NZ. The nuclear DNA of this frequent genotype is closely related to that of genotypes in SA and Aus sharing the same Eurasian-related haplotype (Haplotype F) (Fig. 2). The other genotype was found only once in our study, at Lake Tarawera in the North Island, and we could identify neither its origin nor its closest relationships within our sample set, despite it being the same haplotype as the rest of the NZ samples (Haplotype F). The species is spreading effectively in New Zealand with aid from fishing boats and boat trailers^9^, but also naturally via downstream transport of vegetative fragments^7^. Vegetative reproduction is an advantage for introduced species in a novel environment as it enables the species to establish and spread instantly after introduction and this has been found to be positively correlated with invasion success^21^. Field observations suggest that *C. demersum* is reproducing primarily by fragmentation in New Zealand^7^ and seeds have never been observed in the populations (Dr J Clayton, pers. comm.). This is supported by our findings, as the spectrum of genetic distances (Fig. 3b) indicates that the two genotypes found in NZ do not interbreed, and that the population of the most frequent of the two genotypes has been founded by clonal reproduction. The short pairwise genetic distances among the samples of the frequent genotype (mode = 2, Fig. 3) and the decreasing distribution of genetic distances fit the clonal mode of reproduction^22^. In the case of sexually reproducing and outcrossing organisms, a normal distribution of genetic distances is expected^22^, that is, a pattern more similar to the one we detected in DK with a mode of genetic distances around 10. Nevertheless, a larger sample of populations from NZ should be analysed to conclusively address vegetative vs. sexual reproduction and interbreeding within and between the two distantly related genotypes because the study of Les in Wisconsin, USA, showed that sexual recombination can occasionally occur in *C. demersum* populations, despite genetic diversity may be low^23^.

### *C. demersum* invasion in NZ is driven by high phenotypic plasticity

Invasive populations are often found to have lower genetic diversity than the populations in their native ranges due to genetic bottlenecks and founder effect^24,25^. This diminishes the width of environmental conditions under which the introduced populations may thrive, but this can be counteracted by a high level of plasticity in the introduced populations^2,26^. Populations harbouring inherently high phenotypic plasticity can hypothetically inhabit a diverse range of new localities without undergoing genetic adaptation through natural selection. Phenotypic plasticity has therefore been proposed as a factor contributing to the development of invasiveness in plants^17,27–33^.

In two earlier studies, we explored the phenotypic plasticity of a population in New Zealand compared to a population from within the native range (DK). From the results in the present study we can conclude, that the previously studied population from NZ belongs to the most frequent genotype found in New Zealand. The NZ population was shown to have a higher level of phenotypic plasticity in photosynthesis and relative growth rate in response to temperature and nitrogen concentration in the water compared to the native population^34,35^. The low levels of genetic diversity in *C. demersum* in New Zealand detected in this study may therefore be overcome by high inherent phenotypic plasticity in the present genotypes, as also shown for other aquatic macrophytes^32,36^.

### More *Ceratophyllum* species in New Zealand?

The genus *Ceratophyllum* includes at least six species of which only *C. demersum* is cosmopolitan, whereas the other species have restricted distribution ranges^37^. The other *Ceratophyllum* species are allopatric among each other, but sympatric with *C. demersum*, suggesting that hybridization opportunities might have occurred for *C. demersum* ^37^. Furthermore, hybridization can stimulate the evolution of invasiveness^38^ and has occurred frequently in similar invasive aquatic plants, such as the invasive watermilfoils *Myriophyllum spicatum x sibiricum* ^39^ and *Myriophyllum heterophyllum x hippuroides* ^19^ in North America. There is no evidence of hybrids from our study. However, the high genetic distances between the two NZ genotypes comparable to those between the *C. demersum* and C. *submersum* populations in the native range rise a suspicion that hybrids may exist (Fig. 3). Such distances may be due to the occurrence of two *Ceratophyllum* taxa or an introgressed taxon in our sample set. Only a study of nuclear DNA variation can confirm or rule out the hypothesis of two taxa.

### Possible cryptic invasion in SA and Aus

The introduction of conspecific genotypes may hide cryptic invasions in regions where the species is native^10,40^. In our study the populations in SA and Aus have high AFLP genetic identity to the genotype that is most frequent in the invasive population in NZ (Table 3, Fig. 2). The SA and Aus genotypes also share the same haplotype and a vigorous growth and similar invasive behaviour (J Coetzee and Dr T Bickel, pers. comm.). However, in SA and Aus, *C. demersum* is not classified as “invasive”, as this term by definition is restricted to non-native, introduced species^41^. In the sampled regions in SA and Aus there are also native populations and the introduction of a new haplotype might have been unobserved. *C. demersum* is still sold as an aquarium plant in SA and Aus, which may have been the source of a recent introduction in these countries. Strikingly, the genetic structure of the Eurasian haplotype (Haplotype F) occurring in the US, NZ, Aus and SA is almost identical to that of the invasive haplotype M of *Phragmites australis* (Cav.) Trin ex Steudel (common reed) in North America^42^, which can serve as a reference of a recent cryptic invasion whose genetic dynamics have been exhaustively studied and documented^10,42–46^. A case of convergent evolution in NZ, Aus and SA cannot be ruled out; however, it would be very unlikely that the same mutations (in the chloroplast and in the nuclear DNA) had occurred independently in three disjunct ranges. In the case of independent mutations we would also expect diverging alleles among the populations of the three continents due to allopatric evolution, and an increase in gene diversity when the genotypes of the three populations are considered together. In our study, the genotypes in NZ, Aus and SA shared the same cpDNA sequences and diverged in 2 to 11 AFLP fragments, gene diversity did not increase significantly when we merged the Aus, SA and NZ genotypes and was about half of that found in the Danish population (Table 3). This is in contrast with local speciation processes and supports the hypothesis of a cryptic invasion.

## Materials and Methods

### Plant material

**Figure 4:**
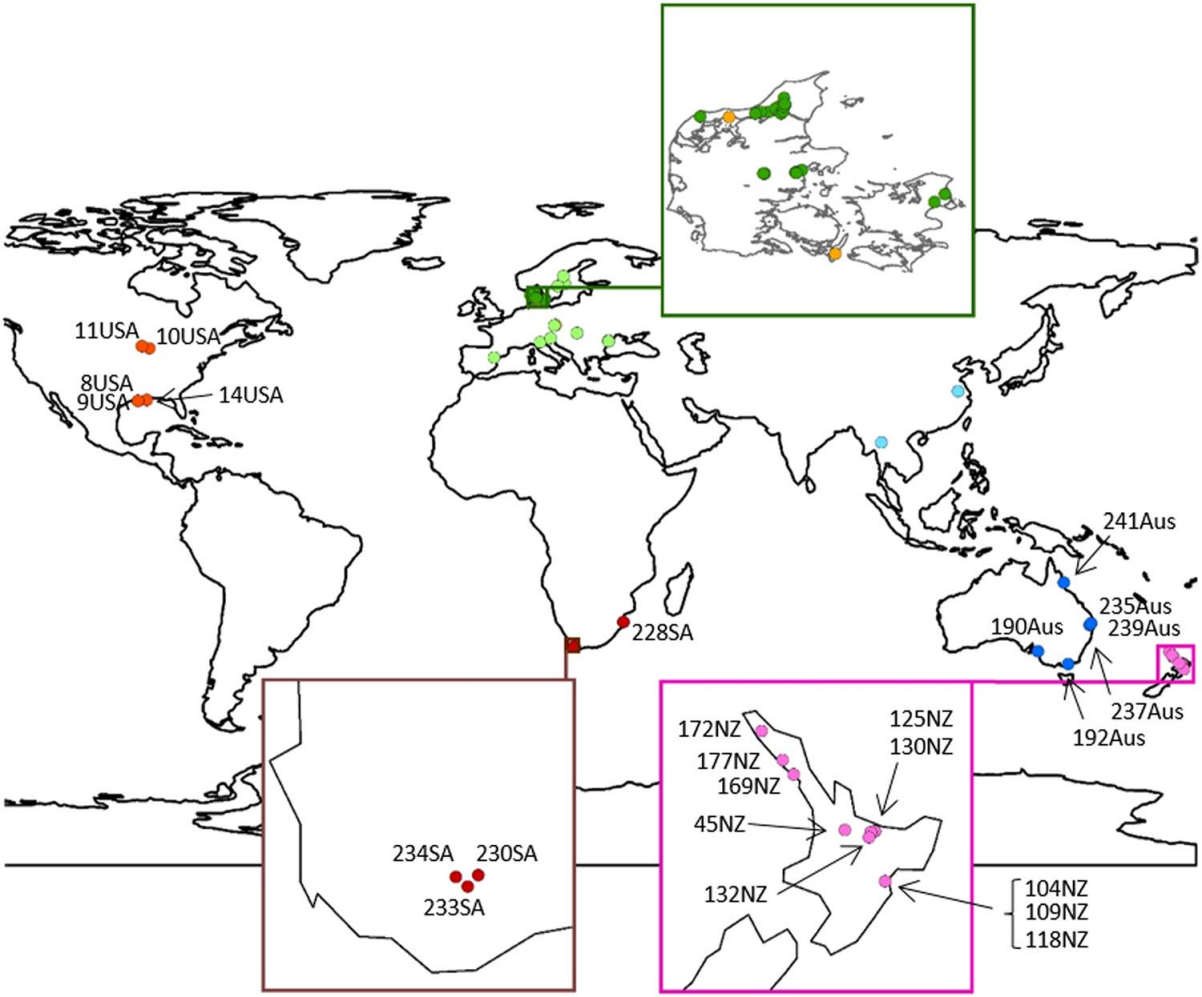
Sampling locations for *C. demersum*: Europe (light green), Denmark (dark green), Asia (light blue), Australia (dark blue), New Zealand (purple), South Africa (dark red), and USA (red), and for *C. submersum* (orange). The samples are placed according to coordinates determined by the collectors. Individual sample numbers have been added where they were relevant. The maps were produced using the computer program ArcGIS 10.5 R-ESRI (www.esri.com).

Shoots of *C. demersum* were collected by colleagues and botanists in geographically widespread locations covering as much of the distribution range of the species as possible, and include both native and introduced populations (Fig. 4). A total of 65 samples from different locations were included in the analyses. We also collected samples of *C. submersum* in Denmark and in the Czech Republic to use as an outgroup for our phylogeography. We checked taxonomic leaf traits of the samples received in order to confirm the species identification made by the collectors. *C. demersum* has dichotomous leaves forked 1–2 times, into 2–4 leaf segments and *C. submersum* leaves are most often forked 2–3 times, producing 3–8 leaf segments^47^.

### DNA Extractions

DNA was extracted from apical parts of shoots dried with silica gel using the E.Z.N.A.^TM^ SP Plant DNA Kit (Omega Bio-Tek Inc., Norcross, Georgia, USA). The protocol was modified for dried samples as suggested by Lambertini *et al*.^42^ to include grinding of the sample in liquid nitrogen and a small amount of autoclaved sand prior to addition of extraction buffer. In addition, incubation at 65 °C was prolonged to 60 min and incubation on ice was 30 min.

### AFLP

The AFLP genotyping was performed using a slightly modified protocol according to Vos *et al*.^48^. Several primer combinations were initially tested on a subset of samples including the most geographically diverse samples to identify primer combinations that produced an adequate number of well separated, clearly polymorphic and reproducible fragments. The full set of samples were analysed using two sets of primers, based on the number of well separated, clear polymorphic fragments: (1) E-CAGcy (5′-GACTGCGTACCAATTCCAG-3′) + M-CAG (5′-GATGAGTCCTGAGTAACAG-3′) and (2) E-CGTcy (5′-GACTGCGTACCAATTCCGT-3′) + M-AGG (5′-GATGAGTCCTGAGTAAAGG-3′). Primers were 6-FAM labelled. Restriction digestion and adapter ligation were performed simultaneous in a 20 µL reaction on approx. 20 ng of genomic DNA, using 1.25 U of each of the restriction enzymes EcoRI and MseI and 0.3 U T4 DNA ligase to ligate 1.25 pmol EcoRI and 12.5 pmol MseI double-stranded nucleotide adapters. We incubated the restriction and ligation reaction in a thermal cycler (Gradient Cycler, Bio-rad Laboratories, Hercules, California, USA) at 37 °C for 4 h followed by a 0.1 °C/s decrease to 16 °C for 2 h and finally 10 min at 70 °C. The restricted DNA was diluted four times prior to preamplification. One-nucleotide selective primers were used in the preamplification, performed with the program: 20 cycles of 94 °C for 30 s, 56 °C for 60 s, 72 °C for 60 s. The preamplification product was diluted 10 times prior to selective amplification. For the selective amplification the program was: 94 °C for 30 s, 65 °C for 30 s (decreased by 0.7 °C/cycle for the subsequent 12 cycles) and 72 °C for 60 s, followed by 23 cycles at 94 °C for 30 s, 56 °C for 30 s and 72 °C for 60 s. The PCR product from the selective amplification was diluted 20 times prior to electrophoresis. The AFLPs were run in an ABI sequencer with an internal ladder in each sample containing 16 fragments ranging in size from 35 bp to 500 bp.

### DNA sequencing

We downloaded all *C. demersum* sequences from GenBank and tested variation in 13 chloroplast regions: trnQ-rps16^49^, trnT-trnL^50^, trnL-trnF^50^, matK^51^, rbcL gene^52,53^, rbcL–psaI^54^, rpl16^55^, rpl32^49^ and the two nuclear internal transcribed spacers of nuclear ribosomal DNA (ITS**)** ^56^ in a subset of intercontinental samples. Given the low intraspecific variation in the published and tested sequences, we also screened variable regions within the chloroplast genome sequenced by Moore *et al*.^57^ (accession no. EF614270.1, GI: 148508422) and designed specific primers in two non-coding regions rich in microsatellites (Cdemex 1 and 2).

Based on the variation in the subset of samples, we chose to include two regions in the analysis, which showed both substitutions and variation at the microsatellite loci. We chose the *rps16* gene encoding the *S16*′ protein, which is a component of the 40 S ribosomal subunit and is amplified by forward primer (rps16F) 5′-GTGGTAGAAAGCAACGTGCGACTT-3′ and reverse primer (rps16R) 5′-TCGGGATCGAACATCAATTGCAAC-3′^58^ and a microsatellite rich region of the cpDNA amplified by the specifically designed forward primer (Cdemex 1 F) 5′-CTATTGACCCCAAGTTCCCT-3′ and reverse primer (Cdemex 1 R) 5′-CAGGCCGTATCGACGGAAAG-3′.

The mastermix for one amplification reaction included 10 ng DNA, 5 µl Red Taq DNA Polymerase 2X Mastermix (VWR – Bie & Berntsen, Herlev, Denmark), 5 pmol of forward and reverse primer, sterile water was added to reach a final volume of 10 µl. PCR amplification was run with an initial step of 3 min at 95 °C, followed by 40 cycles of 95 °C for 30 s, 52 °C for 40 s, 72 °C for 1 min and terminated by a final step of 72 °C for 7 min. Subsequently, 3 µl of PCR products were run on a 1% agarose gel with EtBr for approx. 1 hour (120 V) to check amplification quality and quantity. Sequencing with both forward and reverse primers was performed at GATC Biotech AG, Köln, Germany. All different haplotypes obtained by the cpDNA sequencing were deposited in GenBank.

### Data analysis

#### AFLPs

Genious R6 ver. 6.0.6 (Biomatters Ldt., Auckland, New Zealand) was used to analyse the AFLP data. The internal ladder peaks were aligned and the resulting aligned chromatograms were scored manually for the presence/absence of peaks of equal size.

For the first primer combination we scored fragments in the range between 70 to 300 bp in size. For the second primer combination we scored fragments between 40 and 400 bp. In total we scored 104 polymorphic fragments including both *C. demersum* and *C. submersum*. The DNA was extracted a second time from samples showing diverging chromatograms. The subset of samples used for primer screening was amplified multiple times to ensure reproducibility of the scored peaks. We removed genetically identical AFLP profile samples which were collected from the same locations. We entered question marks for uncertain peaks, either because of low intensity or uncertain size.

#### cpDNA sequences

The sequences were aligned with the program Geneious R6 ver. 6.0.6. To avoid overestimation of the number of mutations associated with mononucleotide repeats we coded repeat motifs (microsatellites) as multistate characters^59^. Hereby, differences due to the number of repeats were counted as one character, independently of their number. Furthermore, indels were coded with two states (presence = 1, absence = 0). The final matrix for the two sequences combined contained 1518 characters. There were 9 substitutions and 5 variable microsatellite loci in total among the *C. demersum* samples. Because of possible DNA slippage at microsatellite loci, the data set was analysed with and without microsatellite repeat motifs, and haplotypes were defined only by substitutions.

The resulting AFLP and cpDNA sequencing results were analysed separately. The cpDNA sequences were analysed with PAUP, ver. 4.0b10 (Phylogenetic Analysis Using Parsimony, Swofford). The data were subject to a jackknife analysis with 37% character deletion, “full heuristic search” and 1000 replicates^60^ and the trees were rooted with five samples of *C. submersum* as a functional outgroup. The consensus tree obtained from the cpDNA sequence matrix was compared with that obtained by the same matrix of cpDNA sequence data, but omitting the microsatellite variation.

The AFLP binary matrix was analysed in a median-joining network with the program Network ver. 4.6.1.1. Phylogenetic Network Constructions (Fluxus Technologies Ltd., Clare, Suffolk, UK) which combines parsimony with minimum spanning tree. The data from the AFLPs were also used to calculate pairwise Nei’s unbiased genetic identities^61^ among geographic regions with GenAlEx ver. 6.5^62^ and Euclidean pairwise genetic distances (i.e pairwise no. of changes). Euclidean genetic distances were used to construct the spectrum of genetic distances and assess reproduction mode of the NZ population. A normal distribution of genetic distance frequencies indicates sexual reproduction, whereas a decreasing curve of genetic distances peaking close to zero is more likely due to clonal reproduction and somatic mutations^22^, and/or artefactual AFLP polymorphisms. We compared the NZ spectrum of genetic distance with that of a native population in Denmark (DK). We included *C. demersum* and *C. submersum* populations in the DK spectrum in order to have a reference of genetic distance patterns within the interbreeding population of *C. demersum* and between the non-interbreeding populations of *C. demersum* and *C. submersum*.

We also calculated AFLP gene diversity (h) (or expected heterozygosity) within the interbreeding Danish population and the introduced population in NZ, and within haplotypes and populations at different geographic scales, in order to identify populations of closely related genotypes.

The datasets generated during and/or analysed during the current study are available from the corresponding author on reasonable request.

## Electronic supplementary material

Supplementary Information
