# Supplementary material for: Phylogeography reveals a potential cryptic invasion in the Southern Hemisphere of Ceratophyllum demersum, New Zealand’s worst invasive macrophyte
Source: Sci Rep. 2017 Nov 29;7:16569. doi: 10.1038/s41598-017-16712-8 (PMC5707378; doi:10.1038/s41598-017-16712-8)
Supplement: Supplementary file 1 — Supplementary Information [file 41598_2017_16712_MOESM1_ESM.pdf]

Phylogeography reveals a potential cryptic invasion in the Southern Hemisphere of Ceratophyllum demersum, New Zealand's worst invasive macrophyte

Benita Hyldgaard[1, 2,] *, Carla Lambertini[1], Hans Brix[1]

Supplementary Table S1 GenBank accession no. and geographic origin of the sequenced samples. Cdemex1 (non-coding region, rich in microsatellites) and *rps16* (gene encoding the *S16'* protein) are in the chloroplast genome. Information about primers can be found in the Materials and Method section.

				**GenBank accession no.**	
Country	Region	Sample no.	Haplotype	Cdemex1	rps16
USA	Louisiana	8USA	A	KJ093282	KJ093291
USA	Wisconsin (Lake Monona)	10USA	A	KJ093283	KJ093292
USA	Wisconsin (LaCrosse)	11USA	A	KJ093284	KJ093293
Denmark	East-Jutland (Risskov)	36DK	E	KJ093285	KJ093294
Italy	Trieste (Gropada)	63I	G	KJ093286	KJ093295
Thailand	Mekong (Chiang Mai)	69Th	C	KJ093287	KJ093296
Denmark	North-Jutland (Vejlerne)	86DK	E		KJ093297
New Zealand	North Island (Taipo stream)	182NZ	F		KJ093298
South Africa	Northen KwaZulu Natal (Lake Sibaya)	228SA	D	KJ093288	KJ093299
Australia	North Queensland (Mulgrave River)	241Aus	B	KJ093289	KJ093300
C. Submersum (Denmark)	East-Jutland (Kasted)	208DKSub		KJ093290	KJ093301

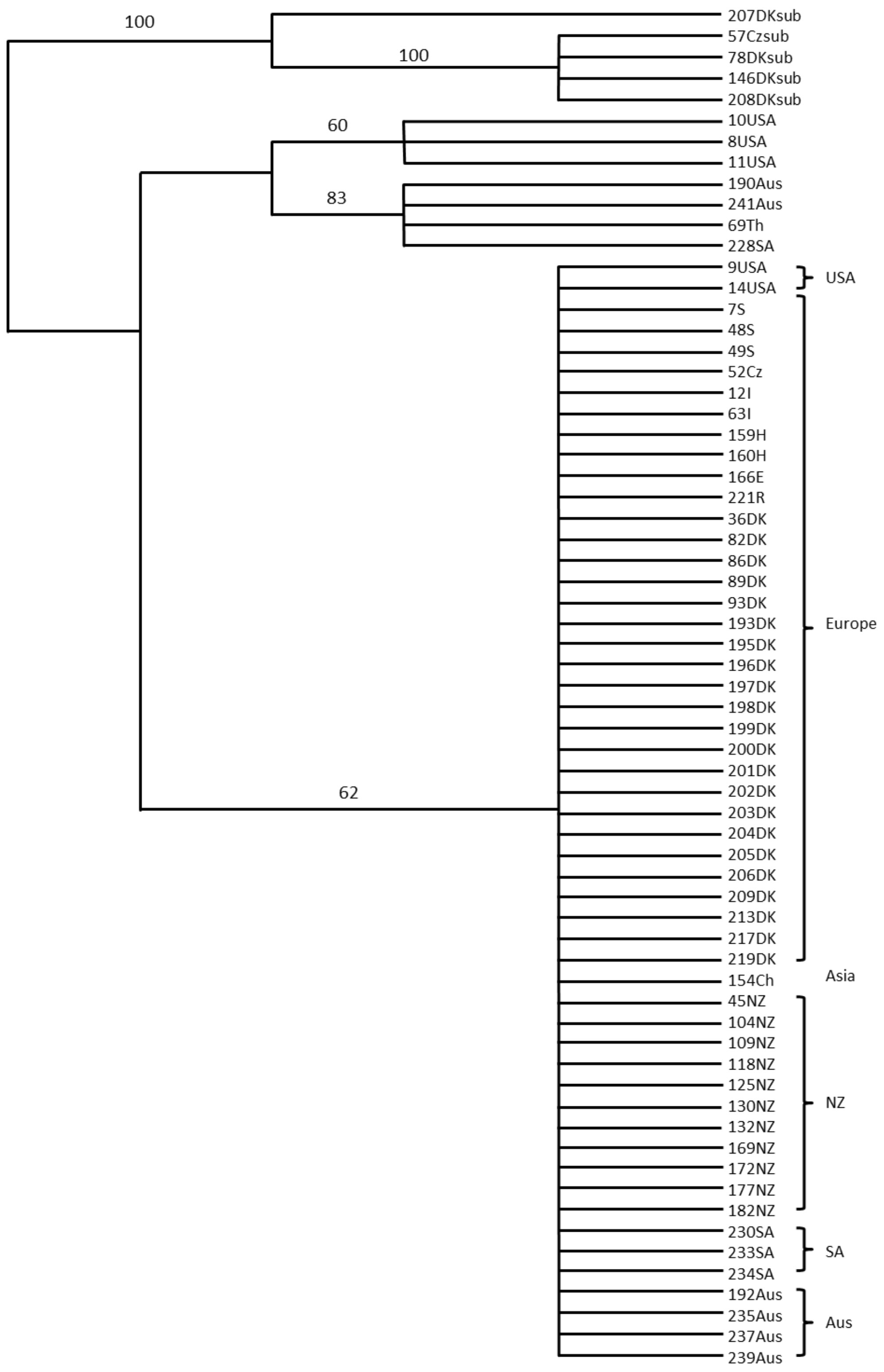

Supplementary Fig. S1 Consensus tree calculated from Cdemex1 and rps16 sequences (as in Fig. 2) with 37% Jackknife after removing microsatellite loci from the analysis
